# The metacognition of auditory distraction: Judgments about the effects of deviating and changing auditory distractors on cognitive performance

**DOI:** 10.3758/s13421-021-01200-2

**Published:** 2021-07-13

**Authors:** Raoul Bell, Laura Mieth, Jan Philipp Röer, Axel Buchner

**Affiliations:** 1 Department of Experimental Psychology, Heinrich Heine University Düsseldorf, 40225 Düsseldorf, Germany; 2 Department of Psychology and Psychotherapy, Witten/Herdecke University, Witten, Germany

**Keywords:** Metacognition, Meta-attention, Judgments of distraction, Auditory distraction, Irrelevant sound effect

## Abstract

The duplex-mechanism account of auditory distraction has been extended to predict that people should have metacognitive awareness of the disruptive effect of auditory deviants on cognitive performance but little to no such awareness of the disruptive effect of changing-state relative to steady-state auditory distractors. To test this prediction, we assessed different types of metacognitive judgments about the disruptive effects of auditory-deviant, changing-state, and steady-state distractor sequences on serial recall. In a questionnaire, participants read about an irrelevant-speech experiment and were asked to provide metacognitive beliefs about how serial-recall performance would be affected by the different types of distractors. Another sample of participants heard the auditory distractors before predicting how their own serial-recall performance would suffer or benefit from the distractors. After participants had experienced the disruptive effects of the distractor sequences first hand, they were asked to make episodic retrospective judgments about how they thought the distractor sequences had affected their performance. The results consistently show that people are, on average, well aware of the greater disruptive effect of deviant and changing-state relative to steady-state distractors. Irrespective of condition, prospective and retrospective judgments of distraction were poor predictors of the individual susceptibility to distraction. These findings suggest that phenomena of auditory distraction cannot be categorized in two separate classes based on metacognitive awareness.

## Introduction

While metacognitive judgments about to-be-learned stimuli (judgments of learning) have received much attention for some decades (e.g., Begg et al., 1989; Koriat, 1997) and still do so (e.g., Besken & Mulligan, 2014; Frank & Kuhlmann, 2017; Schaper et al., 2019; Undorf & Erdfelder, 2015), metacognitive judgments about to-be-ignored stimuli (judgments of distraction) have received considerably less attention (but see Ellermeier & Zimmer, 1997; Hanczakowski et al., 2017, 2018; Röer et al., 2017). However, metacognitive judgments about the effects of task-irrelevant stimuli on performance are of applied relevance because they have a strong influence on how people design their work and learning environments. Assuming that people hold the belief that certain types of distractors have disruptive effects on their performance while others do not affect their performance or have only minor effects, they will take countermeasures against some sources of distraction (to which they ascribe disruptive effects) but not against others (to which they ascribe no or negligible disruptive effects). This is problematic if people’s metacognitive judgments of the distracting effects of task-irrelevant stimuli on performance do not correspond to the actual disruptive effects of these stimuli. If research shows that people systematically underestimate the effects of some sources of distraction on cognitive performance, then it will be particularly important to raise public awareness for these hidden causes of performance disruption. Such metacognitive failures may be more likely under some conditions than others. For instance, people may well be aware of the fact that their focus of attention is drawn to a source of distraction. By contrast, they may have comparatively little awareness of the interference among automatic processes operating outside of the focus of attention. It thus seems possible that metacognitive judgments differ between different classes of distraction depending on the degree to which focal attention is involved. In addition to its applied implications, research on metacognitive judgments about task-irrelevant stimuli can shed light on the causes of distraction and help to refine relevant theories in this area of research.

While some pioneering studies have focused on metacognitive convictions about the disruptive effects of naturalistic sounds such as speech and music on performance (Alley & Greene, 2008; Ellermeier & Zimmer, 1997; Perham & Vizard, 2011; Röer et al., 2017; Schlittmeier et al., 2008), there is as yet surprisingly little knowledge about people’s metacognitive awareness of basic phenomena of auditory distraction. Specifically, the changing-state effect (Jones et al., 1993) and the auditory-deviant effect (Hughes et al., 2005) are seen as key signature findings of auditory distraction (Hughes, 2014) and are among the list of benchmark findings that working memory models should be able to explain (Oberauer et al., 2018). Both effects are examined using serial-recall tasks in which sequences of digits, letters, or words have to be immediately recalled in their correct serial order. Auditory distractors have to be ignored during the presentation of the to-be-remembered sequence or during the memorization of the sequence in a short retention interval. The disruptive potential of the auditory input is primarily determined by changes in the to-be-ignored auditory stream (for a review, see Ellermeier & Zimmer, 2014). Three types of distractor sequences are usually contrasted with each other. *Steady-state sequences* consist of repeated distractors (e.g., A A A A A A A A A). *Changing-state sequences* consist of different distractors (e.g., A B C D E F G H I). *Auditory-deviant sequences* contain a distractor that deviates from the rest of the sequence (e.g., A A A A A B A A A). The *changing-state effect* refers to the robust finding that changing-state sequences disrupt serial recall more than steady-state sequences (Campbell et al., 2002; Jones et al., 1993). The *auditory-deviant effect* refers to the finding that auditory-deviant sequences disrupt serial recall more than steady-state sequences (Vachon et al., 2017). Steady-state sequences themselves have less of an effect on performance than changing-state or auditory-deviant sequences, but their impact on performance is not zero. When sample sizes are reasonably large, it can be robustly observed that steady-state sequences disrupt serial recall relative to a quiet control condition (Bell, Röer, et al., 2019a).

The *duplex-mechanism account of auditory distraction* (Hughes, 2014) postulates that the changing-state effect and the auditory-deviant effect are completely dissociated from each other because they represent two fundamentally different forms of distraction: interference-by-process (Type I) and attentional diversion (Type II). This distinction can be applied to different kinds of phenomena of distraction, but the changing-state effect and the auditory-deviant effect are considered the prototypes of interference-by-process and attentional diversion, respectively (Hughes et al., 2013; Hughes & Marsh, 2019; Marsh et al., 2020). Even though the duplex-mechanism account does not include explicit references to dual-process theories in other areas of research, the characteristics that are postulated to distinguish between the two classes of distraction (amenability to cognitive control, domain specificity, dependence on general cognitive resources, access to awareness), the terminology (“Type I” and “Type II” distraction; Hughes, 2014, p. 30f) and the resulting research program (searching for dissociations to classify empirical phenomena into one of the two classes) align well with dual-process theories in other areas of research that postulate fundamental differences between two cognitive systems (Keren & Schul, 2009).

Table 1 lists the properties that have been proposed so far to be diagnostic of interference-by-process as opposed to attentional diversion. It has, for example, been postulated that interference-by-process occurs obligatorily whereas attentional diversion is under cognitive control (Hughes et al., 2013; Hughes & Marsh, 2019, 2020; Marsh et al., 2018, 2020). Interference-by-process is to be predominantly *automatic* in the sense that it is rooted in the pre-attentional processing of the sounds and thus unaffected by task engagement. Attentional diversion is to be predominantly *controlled* in the sense that the auditory-deviant effect is assumed to depend on a tradeoff in the allocation of attentional resources to the task-relevant visual and the nominally to-be-ignored auditory stimuli, implying that the auditory-deviant effect is eliminated or substantially reduced when task engagement is high (Hughes et al., 2013; Hughes & Marsh, 2019; Marsh et al., 2018, 2020; Marsh et al., 2020). Furthermore, interference-by-process effects are postulated to be domain-specific (Elliott et al., 2016; Joseph et al., 2018; Marsh et al., 2018), while attentional-diversion effects are assumed to be domain-general (Vachon et al., 2017). Accordingly, only attentional diversion, but not interference-by-process, is assumed to be related to general cognitive resources such as working memory capacity (Hughes et al., 2013; Sörqvist, 2010). Several studies have provided empirical support of these claims, but others have reported findings that are inconsistent with these claims and have offered alternative interpretations of the observed dissociations (e.g., Bell et al., 2013, 2017, in press; Körner et al., 2017, 2019; Röer, Bell, & Buchner, 2015), so that the robustness and meaning of the empirical dissociations remains a matter of debate. Specifically, within the *unitary attentional model* originating in the embedded-processes model (Cowan, 1995) the changing-state effect and the auditory-deviant effect are not conceptualized as two completely dissociated processes. Instead, both effects are attributed to the interplay between orienting reactions and their adaptation (Bell, Röer, et al., 2019b).

**Table 1:** Properties of Type-I auditory distraction (interference-by-process) and Type-II auditory distraction (attentional diversion) that have so far been proposed in the literature (e.g., Hughes, 2014; Hughes & Marsh, 2019)

Type I: Interference-by-process	Type II: Attentional diversion
Automatic	Controlled
Domain-specific	Domain-general
Independent of general processing resources	Dependent of general processing resources
Inaccessible to awareness	Accessible to awareness

As the most recent addition to the list in Table 1, the duplex-mechanism account has been extended to include the prediction that people should be aware of attentional diversion but should be unaware of interference-by-process (Hughes & Marsh, 2019). Due to its novelty, the hypothesis of differential awareness of attentional diversion and interference-by-process on performance can be seen as less well established than the other dissociations predicted by the duplex-mechanism account. However, given that the other postulated dissociations are controversial (e.g., Bell et al., in press), it seems attractive to put the predicted dissociation of awareness to an empirical test, the more so as the suggestion that people may have differential awareness of Type-I and Type-II distraction is not just an arbitrary extension of the duplex-mechanism account but in fact quite a natural extension. Within the duplex-mechanism account the auditory-deviant effect is attributed to a diversion of focused attention (Hughes et al., 2013; Hughes & Marsh, 2019; Marsh et al., 2018, 2020). A close link between focused attention and awareness is postulated in most working memory models. For instance, the embedded-processes model (Cowan, 1999) implies that “the information in the focus of attention is the same information that the person is aware of” (p. 89) even though awareness and focused attention may perhaps be dissociated in certain unusual circumstances such as when patients with neurological problems are tested. From the perspective of the duplex-mechanism account, it is thus quite natural to postulate that most individuals should be well aware of the fact that deviant stimuli capture the focus of attention. The changing-state effect, by contrast, is attributed to automatic interference-by-process (Hughes & Marsh, 2019). Definitions of automaticity typically imply that automatic processes are stimulus-driven rather than goal-dependent, do not rely on attentional processes, and occur without awareness (Jacoby et al., 1993). From the perspective of the duplex-mechanism account, it is thus quite natural to postulate that people are largely unaware of the detrimental effects of interference-by-process on their performance (Hughes & Marsh, 2019).

The existing literature has been interpreted by Hughes and Marsh (2019, p. 138) as providing hints for the postulated dissociation in the metacognitive awareness of auditory distraction:

The available evidence suggests that participants show little or no subjective awareness of the degree to which changing-state irrelevant sound disrupts their performance (Ellermeier & Zimmer, 1997), in line with the notion they are not aware of changing-state sound incurring an increase in ‘load’. Indeed, the idea that the participants are unaware of the changing-state effect is in line with the interference-by-process account of that effect but sits uncomfortably with an attentional-diversion based account of the effect. […] A potentially informative extension of the current study, therefore, would be to include measures of participants’ awareness of the interference produced […] by the two forms of auditory distraction. We would expect that participants would be consciously aware of […] disruption by a deviant sound but they would show less awareness of the degree of disruption caused by changing- compared to steady-state sound.

The prediction by Hughes and Marsh (2019) about the relative lack of awareness for the disruptive effects of changing-state relative to auditory-deviant sounds still awaits an empirical test. In the study of Ellermeier and Zimmer (1997) to which Hughes and Marsh (2019, p. 138) refer, the effect of a recorded lecture in a foreign language (Japanese) was compared to the effect of pink noise. Seventy-two participants recalled lists of nine digits that were presented at a rate of 1 Hz while ignoring the auditory distractors. A subgroup of 25 participants was asked to evaluate the effects of the sounds (on a 6-point scale ranging from “will seriously hurt my performance” to “will help considerably”) after they had read the instructions and heard two sample sounds, but before they actually had to ignore the distractors during the serial-recall task. The rating task was repeated after the serial-recall task had been completed. The results showed that participants consistently rated foreign speech as being highly disruptive. Furthermore, they tended to overestimate the effect of pink noise relative to that of continuous speech before participating in the serial-recall task, but were aware that pink noise had only a minor effect on their performance after having completed the task. The subjective judgments were poor predictors of the degree to which participants were actually disrupted by the different types of sounds but their validity improved after first-hand experience of disruptive effects of sounds on serial recall. Importantly, these results are completely inconclusive with respect to a possible dissociation between the changing-state effect and the auditory-deviant effect. First, metacognitive judgments about the disruptive effect of auditory deviants have not been assessed at all, so that it is impossible to evaluate the hypothesis of a dissociation. Second, the classical changing-state effect was not examined as Japanese lectures and pink noise differ from standard changing-state and steady-state sequences, respectively. To draw clear conclusions about the participants’ relative lack of awareness of the disruptive effect of changing-state versus auditory-deviant sounds, the procedure of Ellermeier and Zimmer (1997) has to be adapted to systematically assess participants’ metacognitive judgments about steady-state, auditory-deviant, and changing-state distractor sequences.

In accordance with procedures that have been developed to study metacognitive judgments of learning (e.g., Besken & Mulligan, 2014; Frank & Kuhlmann, 2017; Schaper et al., 2019; Undorf & Erdfelder, 2015), we distinguish between three types of metacognitive assessments of the effect of task-irrelevant sound on performance. *Abstract metacognitive beliefs* (cf. Mueller et al., 2014) are convictions or naïve theories that are independent of, and transcend, the immediate perceptual experience of the stimuli. Here, the beliefs about steady-state, auditory-deviant, and changing-state sequences were assessed by three questions embedded in a more comprehensive survey about metacognitive beliefs on auditory distraction. In this survey, participants read abstract descriptions of the levels of the distractor-sound manipulation without being provided with concrete examples of these distractors so that their judgments about how this type of material may affect serial-recall performance could not be based on the immediate experience of perceptual cues but had to be based on the participants’ preexisting beliefs. *Prospective metacognitive judgments* (cf. Begg et al., 1989) were assessed in an experiment in which steady-state, auditory-deviant, and changing-state sequences were played to the participants who predicted how much they would be affected by each individual distractor sequence when trying to memorize a sequence of digits for serial recall. After participants had actually ignored these distractor sequences while memorizing digits in a serial-recall task, they were asked to provide *retrospective metacognitive judgments* (cf. Frank & Kuhlmann, 2017) about how much they thought their performance had been affected by the different types of sounds. Based on the duplex-mechanism account, it can be postulated that people’s metacognitive judgments should reflect a strong metacognitive awareness of the auditory-deviant effect combined with a relative lack of awareness of the changing-state effect (Hughes & Marsh, 2019). The unitary attentional account (Cowan, 1995) does not predict such a dissociation because it attributes both effects to attentional orienting. The present study thus allows for a novel test of these two competing accounts.

## Metacognitive beliefs

### Method

#### Participants

Participants were recruited on the campus of Heinrich Heine University Düsseldorf. We aimed to recruit about the same number of psychology students and students of other disciplines to be able to check whether expertise in psychology would have an effect on the metacognitive judgments. Prior to data analysis, six data files had to be removed because six people had participated twice. The final sample consisted of 189 participants (143 of whom were female). With this sample size and α = .05, an effect of distractor type of size η_p_^2^ = .10 could be detected with a statistical power of 1 – β = .99. About half of the participants (95) were psychology students while the other half (94) were students from other disciplines. Their age ranged from 17 to 38 years with a mean age of 23 years (*SD* = 4). They all signed written informed consent prior to the start of the experiment. They received course credit or a small monetary compensation.

#### Design, materials, and procedure

At the start of the survey, participants were asked to imagine that they had to perform a serial-recall task that was described to them. They were seated in front of a computer and asked to complete one sample trial of this task. In this sample trial, eight different digits appeared, one after another, in 80 pt Monaco font, in the middle of the screen of the computer that controlled the experiment. The digits were shown for one second each. Participants were instructed to memorize the digits in the order of their appearance on the screen. Immediately after the presentation of the last digit, eight question marks appeared on the screen. The question marks had to be replaced by typing the digits in their correct order using the number pad of the computer’s keyboard. Participants were not allowed to correct their responses or to skip a digit.

**Fig. 1:**
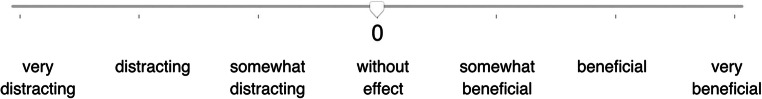
The rating scale on which participants judged the effects of the different types of sounds that were displayed to them. The scale ranged from –100 (*very distracting*) to +100 (*very beneficial*) with 200 increments from one end point to the other

After the sample trial had been completed, participants were informed that they would see verbal descriptions of different types of sounds. They were asked to imagine hearing these sounds during the memorization task and to judge the effect that each type of sound would have on their performance in the serial-recall task. The question “How distracting or beneficial is the following sound for the task?” was displayed at the top of the screen. At the middle of the screen, a specific type of sound (e.g., “A series of different one-syllable words”) was verbally described. At the bottom of the screen, a rating scale ranging from –100 (*very distracting*) to +100 (*very beneficial*) was shown. We did not only label the endpoints of the scale but also intermediate points because this helps participants to understand the meaning of the scale and thus reduces the variability in the interpretation across respondents (Maitland, 2009). Furthermore, we used verbal labels for which a satisfactory degree of inter-individual agreement about their meaning has been empirically determined (Rohrmann, 1978). Participants used the scale’s slider to indicate whether these sounds would have a more or less distracting, a more or less beneficial, or no effect on the task of remembering the digits (Fig. 1).

There were 32 thematic clusters of questions in which participants were asked to judge the distracting effects of different types of auditory distractor features (e.g., familiar vs. unfamiliar music). For each participant, the clusters were presented in a different, randomly determined order, as were the questions within each cluster. Here, we focus on the cluster of questions about the relative disruptive effects of steady-state, auditory-deviant, and changing-state sequences of distractor words. Within this cluster, participants were asked to rate the effects of “The repeated presentation of a one-syllable word,” “A series of repeated one-syllable words with a deviating word in about the middle of the list,” and “A series of different one-syllable words.” Thus, the distractor type independent variable had three levels and participants’ ratings served as the dependent variable.

### Results

Preliminary analyses showed that whether or not participants were enrolled in a psychology program did not significantly affect the metacognitive beliefs about the effects of the different distractor types on serial-recall performance. Therefore, this factor is not included in the analyses reported below.

**Fig. 2:**
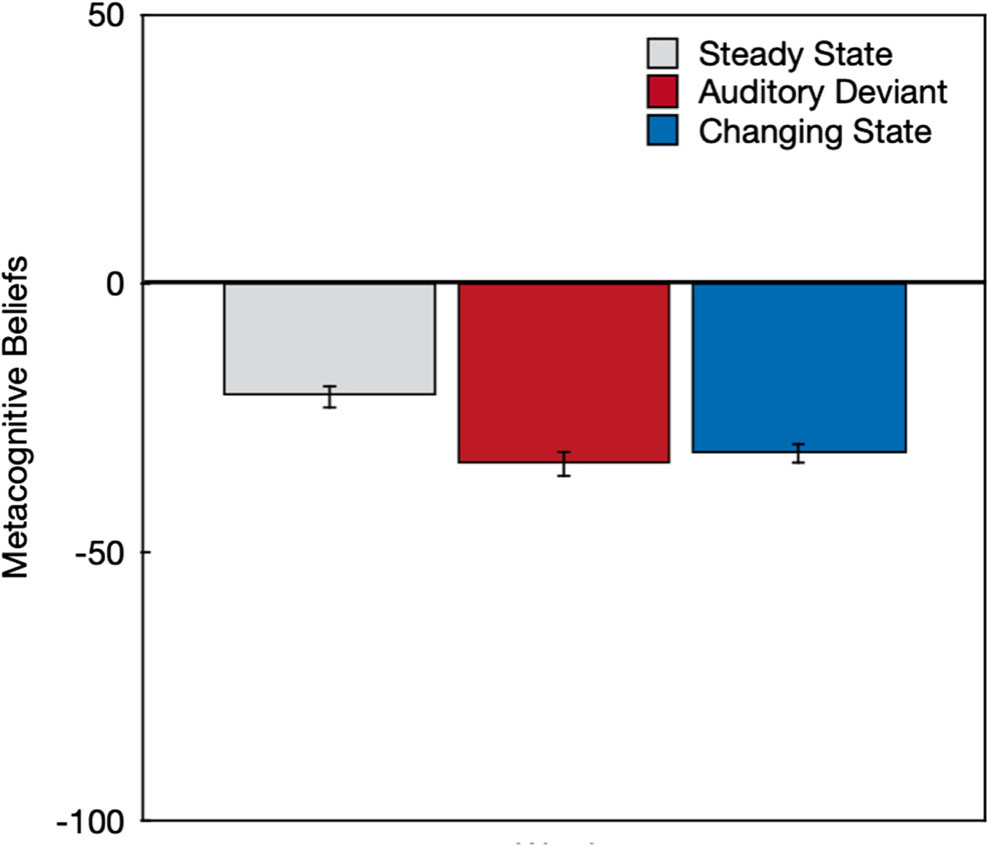
Metacognitive beliefs based on abstract written descriptions of distractor sequences about the effects of steady-state, auditory-deviant, and changing-state sequences on serial-recall performance in terms of the believed effect of the sound sequences on performance on a scale ranging from –100 (*very distracting*) to +100 (*very beneficial*). The error bars represent the standard errors of the means

All sounds were associated with negative ratings (Fig. 2), indicating that, on average, the word sequences described were believed to be disruptive rather than beneficial for serial-recall performance, *F*(1,188) = 256.79, *p* < .001, η_p_^2^ = .58. A repeated-measures analysis using the MANOVA approach as suggested by O'Brien and Kaiser (1985) revealed that people’s metacognitive beliefs about the effects of distractor words on serial-recall performance varied as a function of distractor type, *F*(2,187) = 18.80, *p* < .001, η_p_^2^ = .17. Orthogonal contrasts showed that participants believed steady-state sequences to be less disruptive than auditory-deviant and changing-state sequences, *F*(1,188) = 36.55, *p* < .001, η_p_^2^ = .16. Importantly, they also believed auditory-deviant and changing-state sequences to be equally disruptive, *F*(1,188) = 0.68, *p* = .41, η_p_^2^ < .01.

Separate supplemental analyses showed that participants believed auditory-deviant sequences to be more disruptive than steady-state sequences, *F*(1,188) = 32.13, *p* < .001, η_p_^2^ = .15, which corresponds to the belief in an auditory-deviant effect. They also believed changing-state sequences to be more disruptive than steady-state sequences, *F*(1,188) = 20.78, *p* < .001, η_p_^2^ = .10, which corresponds to the belief in a changing-state effect.

### Online replication

A possible caveat is that it remains to be tested whether the metacognitive beliefs about steady-state, auditory-deviant, and changing-state sequences have been affected by the fact that the questions were embedded in a larger survey about the effects of various types of sounds on cognitive performance. To test this, we performed an online replication of the laboratory survey (involving *N* = 87 participants) in which only the three questions about steady-state, auditory-deviant, and changing-state sequences were included. All of the findings of the original laboratory survey have been fully replicated. A detailed description of the online replication study and its data are available at https://osf.io/zxqy9/.

### Discussion

Metacognitive beliefs were assessed by showing participants abstract descriptions of different types of distractor sequences and asking them to indicate how they believed that these sequences would affect serial-recall performance. Steady-state sequences were ascribed a disruptive effect, but they were consistently rated as less disruptive than both auditory-deviant and changing-state sequences. Auditory-deviant and changing-state sequences were rated to be equally disruptive.

Based on these results, the idea of a *relatively* less pronounced awareness for the disruptive effect of changing-state relative to auditory-deviant sequences cannot be completely rejected. When the actual disruptive effect of auditory-deviant sequences and changing-state sequences are compared in real experiments, the changing-state effect is often much larger than the auditory-deviant effect (e.g., Bell, Mieth, et al., 2019; Hughes et al., 2005; Marois et al., 2019). One could thus argue that, relative to the effect of changing-state sequences, participants’ metacognitive beliefs overestimated the disruptive effect of auditory-deviant sequences somewhat because they believed them to be just as disruptive as changing-state sequences. However, the results are clearly incompatible with the idea that people are *unaware* of the disruptive effect of changing-state sequences as their beliefs were in line with the disruptive nature of both the auditory-deviant effect *and* the changing-state effect. The strong hypothesis that people have little to no awareness of the disruptive effect of changing-state sequences was thus disconfirmed by the results.

However, when Hughes and Marsh (2019) postulated that people should show little or no subjective awareness of changing-state disruption, they referred to prospective and retrospective metacognitive judgments about the disruptive effects of specific stimuli (Ellermeier & Zimmer, 1997). It is therefore interesting to examine how participants judge the effects of distractor sequences when experiencing the distractors’ phenomenological characteristics first-hand. According to cue-based accounts (Koriat, 1997), metacognitive judgments about specific stimuli are not only based on beliefs, but also on the immediate perceptual experience of the stimuli such as their ease-of-processing (e.g., Besken & Mulligan, 2014; Frank & Kuhlmann, 2017; Schaper et al., 2019). Examining experience-based metacognitive judgments is also of interest from an applied perspective because it can be assumed that, in practice, metacognitive judgments about the effects of auditory stimuli on performance are often made in situations in which people are directly exposed to these stimuli.

## Prospective and retrospective metacognitive judgments

### Method

#### Participants

We aimed at collecting data of about 200 participants and continued data collection until the end of the week in which this goal was reached. Before analyzing the data, two data files were deleted because of double participation, and one data file was not correctly saved and thus could not be analyzed. The remaining sample consisted of 213 participants (166 of whom were female) who were recruited on the campus of Heinrich Heine University Düsseldorf. With this sample size and α = .05, an effect of distractor type of size η_p_^2^ = .10 could be detected with a statistical power of 1 – β = .99. About half of the participants (112) studied psychology (103 of them in an undergraduate study program) while the other half (101) did not study psychology. Their age ranged from 17 to 42 years with a mean age of 23 years (*SD* = 4). All participants signed a written informed consent prior to the start of the experiment. They received course credit or a small monetary compensation for participating.

#### Materials

Each auditory word sequence was generated by drawing one-syllable words from the word set {Alm [alp], Elch [moose], Gel [gel], Jod [iodine], Los [lot], Milz [spleen], Ohm [ohm], Schopf [tuft], Steg [plank], Streu [mulch], Tau [dew], Zwist [strife]} (English translation in brackets). The words were spoken by a female voice, recorded with a 44.1 sampling rate in 16-bit format, were normalized and edited to last 600 ms. For each participant, a set of 30 auditory word sequences (ten steady-state, ten auditory-deviant, and ten changing-state sequences) was individually generated. For each steady-state sequence, one of the words was randomly drawn from the word set and repeated nine times. The auditory-deviant sequences were constructed in the same way as the steady-state sequences but the sixth word of the sequence was replaced by a different word from the set (the deviant word). For each changing-state sequence, nine words were randomly drawn from the set with the restriction that successive distractors were always different from each other. The sequences were thus generated in the same way as in previous studies in which robust changing-state effects and auditory-deviant effects have been observed (e.g., Bell, Mieth, et al., 2019). The sound sequences lasted 9 s, implying that the words were played at a rate of one per second. Throughout the whole experiment, the sounds were played at 65 dB(A) Leq.

#### Prospective metacognitive judgments

At the start of the experiment, participants were seated in front of the computer that controlled the experiment and were provided with a sound-insulating headphone. Next they were asked to imagine that they had to perform a serial-recall task that was described to them. They were then asked to complete one sample trial of this task. Participants started the sample trial by pressing the spacebar of the computer’s keyboard. Nine digits were presented, one after another, in 80 pt Monaco font, at the center of the computer’s screen. The digits were shown for 1 s each. Participants were instructed that their task was to memorize the digits in the order of their appearance on the screen. Immediately after the presentation of the last digit, nine question marks appeared on the screen that had to be replaced by typing the digits in their correct order into the number pad of the computer’s keyboard.

After this sample trial had been completed, participants were asked to perform a metacognitive judgment task. Participants were informed that sounds would be played to them over the headphones. They were asked to imagine hearing these sounds while performing the memorization task of which they had just seen a sample trial. In each trial of the judgment task, a button labeled “Play the sound” appeared at the middle of the screen. Upon clicking this button, one of the auditory word sequences was played to them. After the last word of the sequence had been played, the question “How distracting or beneficial is this sound for the task?” appeared at the top of the screen. Participants were asked to indicate, on the rating scale displayed in Fig. 1, whether the auditory word sequence would have a more or less distracting, more or less beneficial, or no effect on their performance in the imagined serial-recall task. Each participant judged the effects of all 30 auditory word sequences (ten steady-state, ten auditory-deviant, and ten changing-state sequences) in a different, randomly determined order.

#### Objective sound effects

After the judgment task, participants were informed that their next task was the serial-recall task they had imagined before. They were asked not to speak the digits out loud. They were told that the words played through the headphones were completely irrelevant for the task at hand and that they would not be asked about the words later in the experiment. They were asked to focus only on the visually presented digits. The serial-recall task consisted of ten quiet, ten steady-state, ten auditory-deviant, and ten changing-state trials that were presented in a random order. Pressing the space bar started the presentation of the sequence of to-be-remembered digits. The distractor words were presented simultaneously with the digits. Immediately after the sequence of target digits had been shown, participants had to recall the digits in the correct order by typing them into the number pad of the computer’s keyboard, thereby replacing nine question marks that were displayed at the center of the screen. Participants were not allowed to correct their responses or to skip a digit.

#### Retrospective metacognitive judgments

After all trials of the serial-recall task had been completed, participants were asked to provide retrospective metacognitive judgments about the effects of the three types of sounds they had heard during the serial-recall task. Participants were first informed about the type of sound whose effect on the task they had to evaluate (e.g., “In some trials you heard a series of repetitions of the same word”). They were then asked “How distracting or beneficial was this type of sound for the serial-recall task?” At the bottom of the screen, a rating scale ranging from –100 (*very distracting*) to +100 (*very beneficial*) was shown. Participants were asked to judge whether the sounds had a more or less distracting, a more or less beneficial, or no effect on their performance in the serial-recall task. Participants were asked about the effect of steady-state, auditory-deviant, and changing-state sequences in a randomly determined order.

#### Open question

In a final open question, participants were asked “Please describe: Why did you judge the sounds as distracting or beneficial in the first phase of the study?” Participants typed their answer in a text box using the computer keyboard.

### Results

#### Prospective metacognitive judgments

**Fig. 3:**
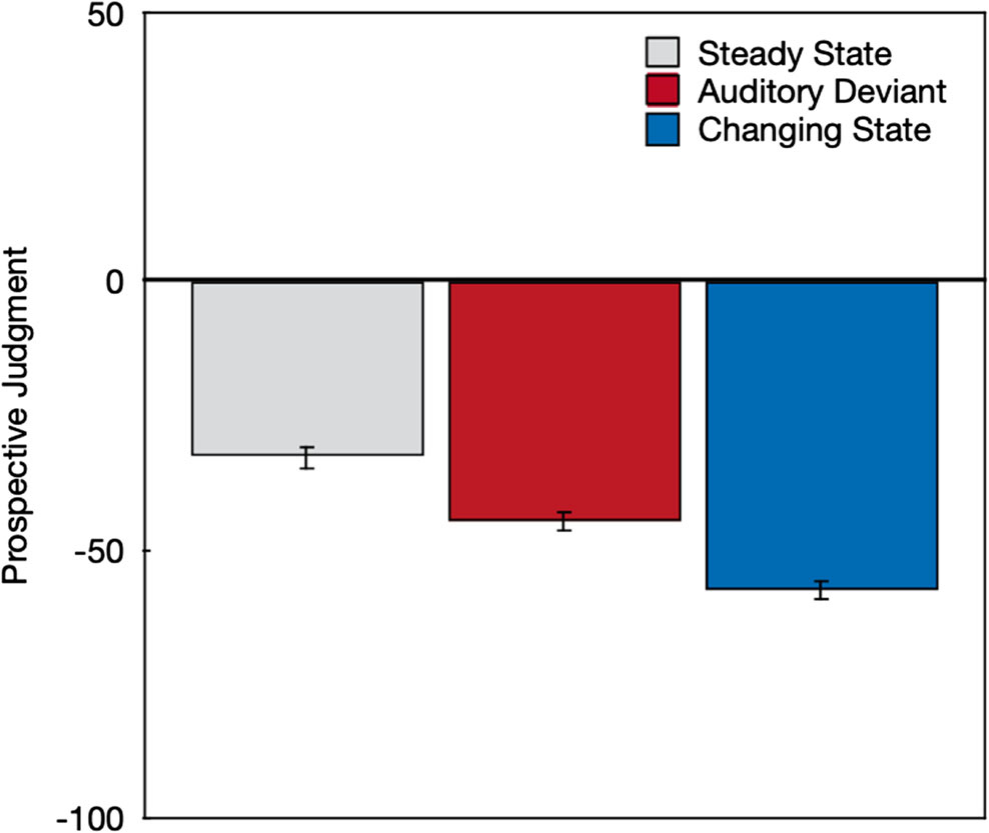
Prospective metacognitive judgments about the effects of specific steady-state, auditory-deviant, and changing-state sequences that were played to participants in terms of the predicted effects of the sound sequences on serial-recall performance on a scale ranging from –100 (*very distracting*) to +100 (*very beneficial*). The error bars represent the standard errors of the means

On average, the sounds were associated with negative ratings (Fig. 3) in the prospective metacognitive judgments, which indicates that, on average, the word sequences that were played to the participants were predicted to be disruptive rather than beneficial to serial-recall performance, *F*(1,212) = 692.82, *p* < .001, η_p_^2^ = .77. Distractor type had a significant effect on the prospective metacognitive judgments, *F*(2,211) = 53.64, *p* < .001, η_p_^2^ = .34. Orthogonal contrasts showed that participants judged steady-state sequences to be less disruptive than auditory-deviant and changing-state sequences, *F*(1,212) = 106.85, *p* < .001, η_p_^2^ = .34. Importantly, they also judged changing-state sequences to be more disruptive than auditory-deviant sequences, *F*(1,212) = 48.09, *p* < .001, η_p_^2^ = .18.

Separate supplemental analyses showed that participants judged auditory-deviant sequences to be more disruptive than steady-state sequences, *F*(1,212) = 67.50, *p* < .001, η_p_^2^ = .24. They also judged changing-state sequences to be more disruptive than steady-state sequences, *F*(1,212) = 102.56, *p* < .001, η_p_^2^ = .33.

#### Objective sound effects

As in previous studies (e.g., Bell, Mieth, et al., 2019), a strict criterion was used to score objective performance in the serial-recall task: Only digits recalled at the correct serial position were scored as correct. To compare the objective performance with the metacognitive judgments about the effect of the sounds on performance, we subtracted performance in the quiet control condition from performance in each condition to compute the measure that captures the objective effect of the sound in mean numbers of digits per trial that were recalled less (or more) when the sounds were played relative to when no sounds were played.

**Fig. 4:**
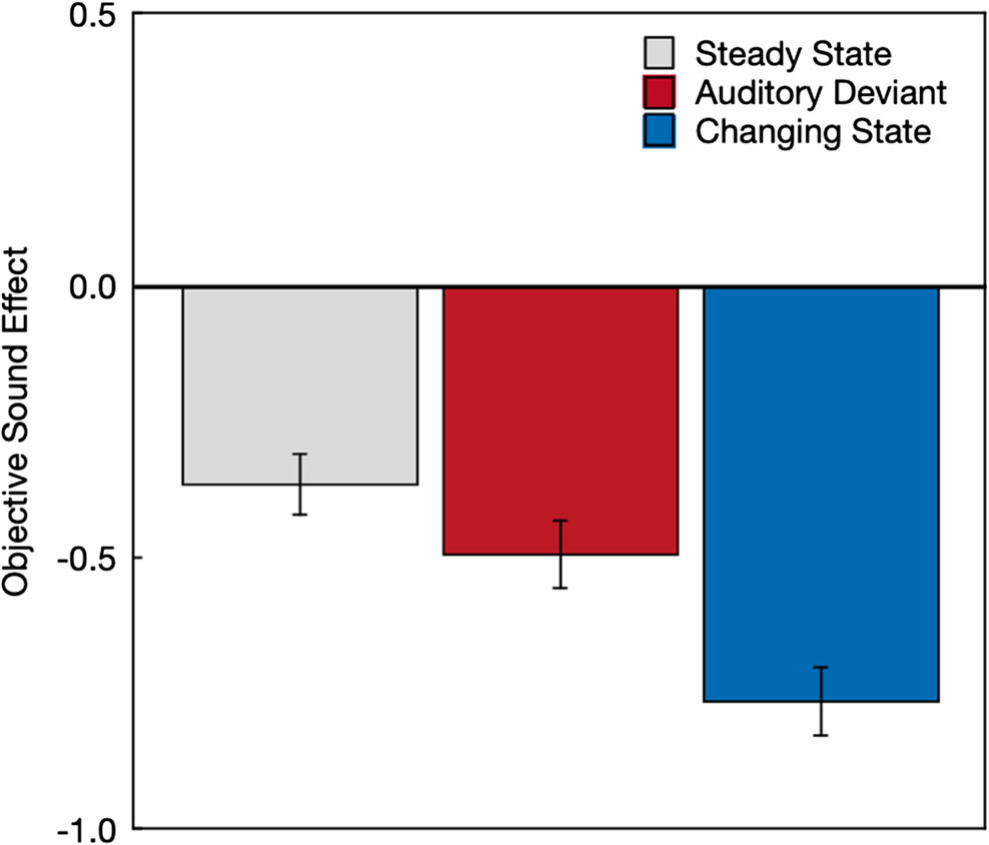
Objective effects of the steady-state, auditory-deviant, and changing-state sequences on serial-recall performance measured by the differences between the distractor conditions and the quiet control condition in mean number of digits recalled per trial. Negative values stand for negative effects of the auditory distractors on performance (fewer digits recalled in the distractor conditions relative to the quiet control condition). The error bars represent the standard errors of the means

All of the effects were in the negative direction (Fig. 4), indicating that, on average, all types of distractor sounds had a negative effect on performance, *F*(1,212) = 105.10, *p* < .001, η_p_^2^ = .33. Distractor type had a significant effect on the objective effects of the sequences on serial-recall performance, *F*(2,211) = 22.32, *p* < .001, η_p_^2^ = .17. Orthogonal contrasts showed that steady-state distractors were less disruptive than auditory-deviant and changing-state sequences, *F*(1,212) = 28.95, *p* < .001, η_p_^2^ = .12. Furthermore, changing-state sequences were more disruptive than auditory-deviant sequences, *F*(1,212) = 18.39, *p* < .001, η_p_^2^ = .08.

Separate supplemental analyses showed that auditory-deviant sequences had a more disruptive effect on serial-recall performance than steady-state sequences, *F*(1,212) = 5.14, *p* = .02, η_p_^2^ = .02, which represents evidence of an auditory-deviant effect. Furthermore, changing-state sequences were more disruptive than steady-state sequences, *F*(1,212) = 44.55, *p* < .001, η_p_^2^ = .17, which represents evidence of a changing-state effect.

#### Retrospective metacognitive judgments

**Fig. 5:**
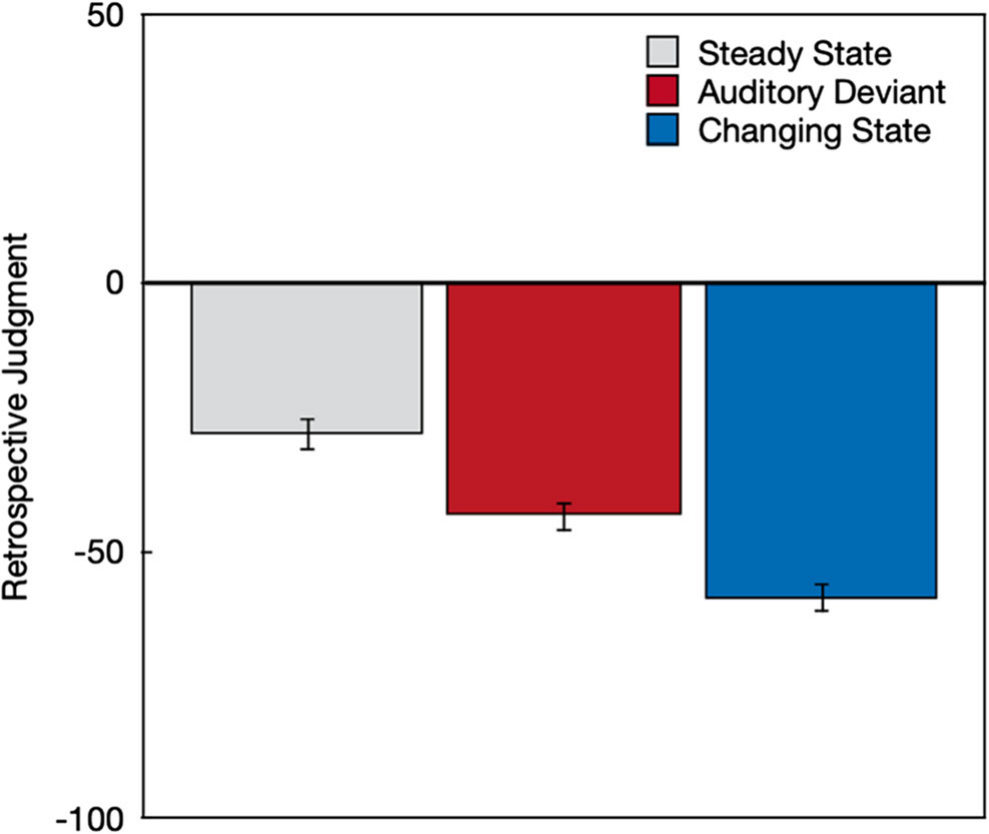
Retrospective judgment about the experienced effects of steady-state, auditory-deviant, and changing-state sequences on serial-recall performance during the preceding serial-recall task on a scale ranging from –100 (*very distracting*) to +100 (*very beneficial*). The error bars represent the standard errors of the means

All sounds were associated with negative retrospective metacognitive judgments (Fig. 5), which indicates that, on average, the described word sequences were rated as having had a disruptive rather than a beneficial effect on cognitive performance, *F*(1,212) = 362.34, *p* < .001, η_p_^2^ = .63. Distractor type had a significant effect on the retrospective metacognitive judgments of the sound effects experienced during the experiment, *F*(2,211) = 42.73, *p* < .001, η_p_^2^ = .29. Orthogonal contrasts showed that participants retrospectively judged steady-state sequences to have been less disruptive than auditory-deviant and changing-state sequences, *F*(1,212) = 74.63, *p* < .001, η_p_^2^ = .26. Importantly, they also retrospectively judged changing-state sequences to have been more disruptive than auditory-deviant sequences, *F*(1,212) = 32.57, *p* < .001, η_p_^2^ = .13.

Separate supplemental analyses showed that participants retrospectively judged auditory-deviant sequences to have been more disruptive to their serial-recall performance than steady-state sequences, *F*(1,212) = 34.02, *p* < .001, η_p_^2^ = .14. They also retrospectively judged changing-state sequences to have been more disruptive than steady-state sequences, *F*(1,212) = 85.78, *p* < .001, η_p_^2^ = .29.

#### Open question

**Fig. 6:**
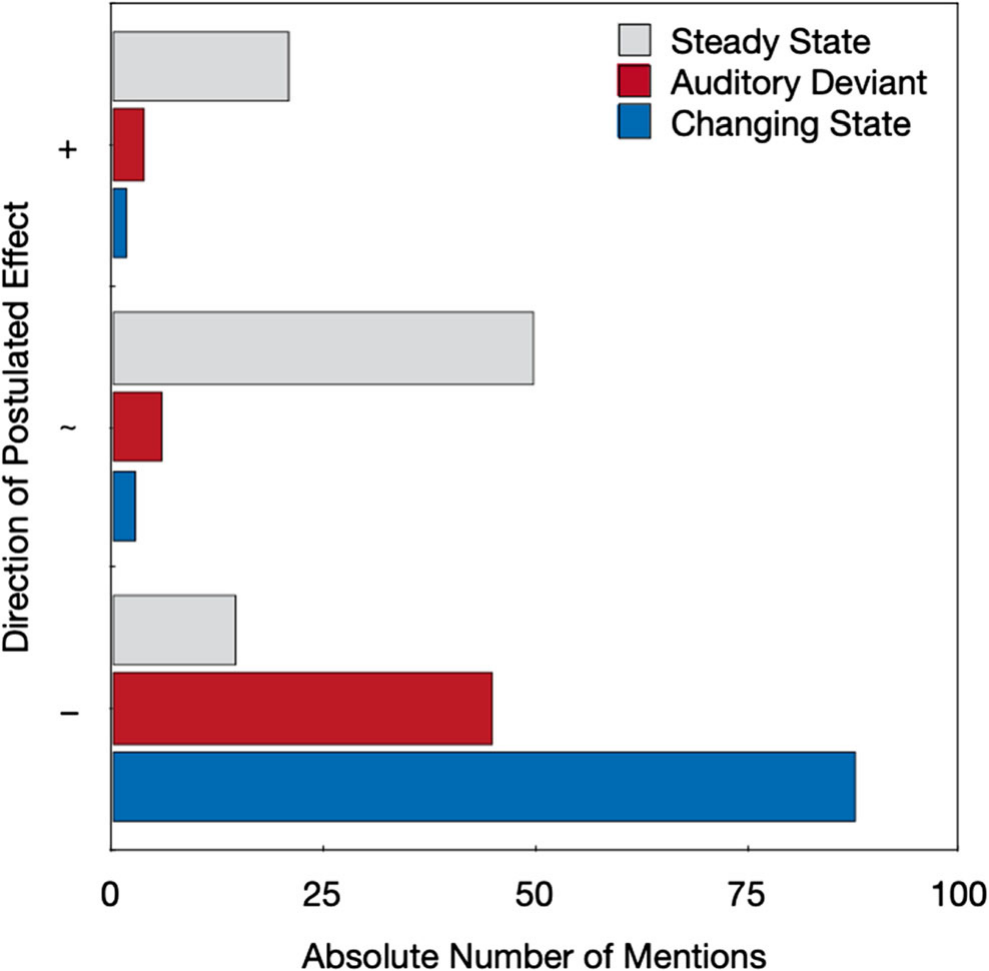
The absolute number of times a specific type of distractor sequence was mentioned to have been beneficial to serial-recall performance (+), not disruptive or less disruptive than other sequences (∼), or disruptive or more disruptive than other sequences (–) in the open question. If, for example, both changing-state as well as auditory-deviant sequences were mentioned as being disruptive by the same participant, both answers were scored in this analysis. Ambiguous answers that referred to steady-state, auditory-deviant, or changing-state sequences, but could not be clearly classified into any of these categories (34 answers in total) are not displayed; neither are answers that refer to other stimulus characteristics

Here, we report only those answers specifically related to the absolute or relative disruptive potential of steady-state, auditory-deviant, and changing-state sequences. Figure 6 displays the number of times a specific type of sequence was mentioned to have been beneficial for serial-recall performance (upper part), not disruptive, or less disruptive than other sequences (middle part), or disruptive or more disruptive than other sequences (lower part). Although steady-state sequences were found to be disruptive or more disruptive than other sequences by a few participants, they were more often mentioned as being less disruptive than the other sequences or even beneficial for performance (e.g., some participants speculated that the monotonous rhythm of the steady-state sequences helped them to memorize the digits). Auditory-deviant sequences were also predominantly mentioned as being disruptive or more disruptive than other sequences, but less frequently than the changing-state sequences. Changing-state sequences were most frequently mentioned as being disruptive or more disruptive than other sequences.

#### Exploratory correlational analyses

**Fig. 7:**
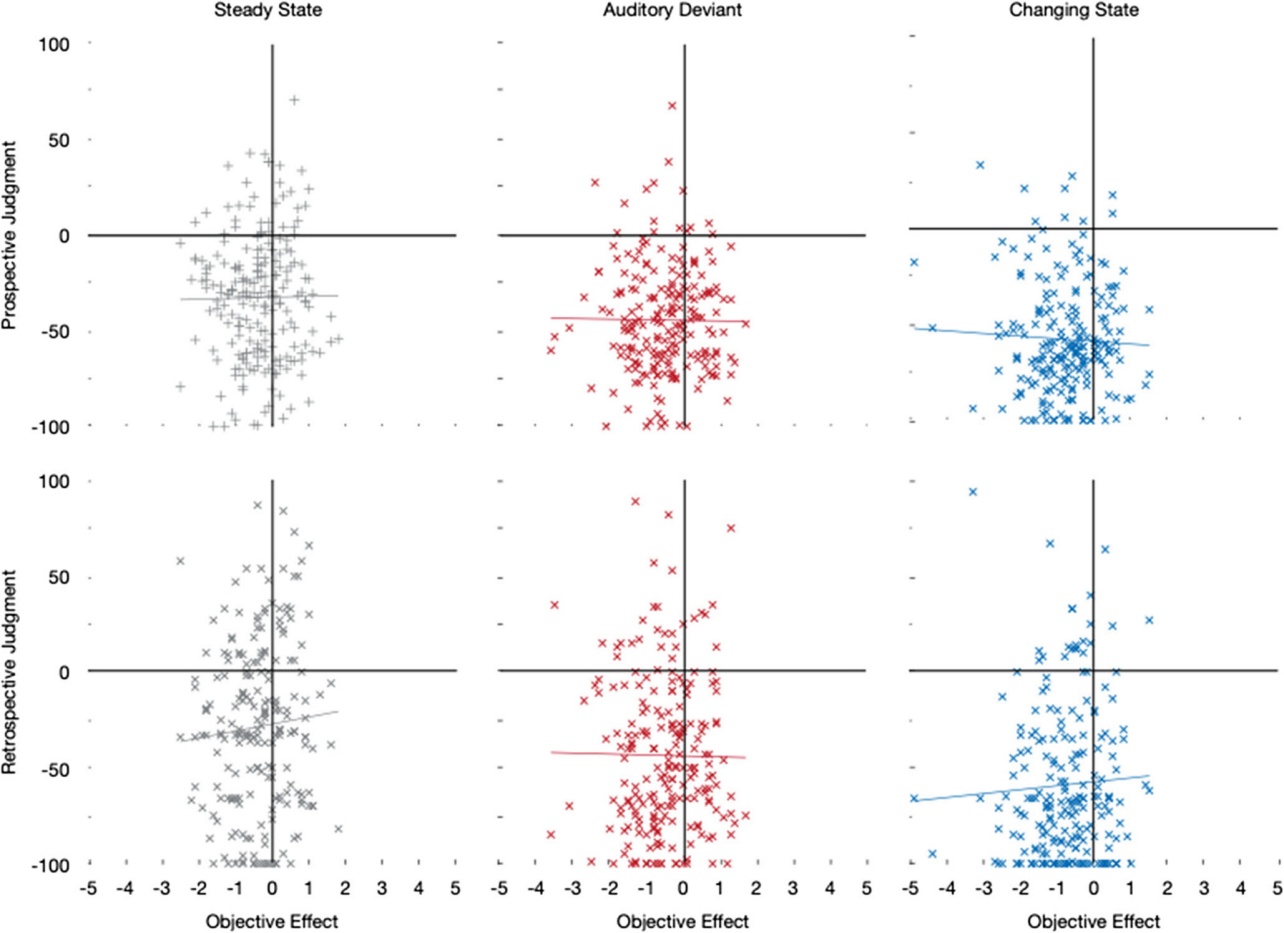
Within-condition relationship between the objective effect of the steady-state, auditory-deviant, and changing-state sequences on serial-recall performance (measured by the difference between the distractor conditions and the quiet control condition in the mean number of digits recalled per trial) and the prospective and retrospective metacognitive judgments of the effects of steady-state, auditory-deviant, and changing-state sequences on performance

In a further exploratory analysis, we analyzed whether those participants who thought to have benefitted from the background sounds actually showed an improvement in performance when listening to the auditory distractors while memorizing the digits. In Figure 7 the objective effect of the steady-state, auditory-deviant, and changing-state sequences on serial-recall performance (measured by the difference between the distractor conditions and the quiet control condition in the mean number of digits recalled per trial) is plotted against the prospective and retrospective judgments of the disruptive potential of the steady-state, auditory-deviant, and changing-state sequences on serial-recall performance. Most of the data points are in the lower left quadrant of each plot, indicating that most participants (correctly) believed the sounds to have a disruptive effect on performance while they were indeed disrupted by the distractor sounds, whereas there are only few data points in the upper right quadrant of each plot, representing participants who believed sounds to have a beneficial effect on performance and indeed showed better performance when sounds were played during memorization than when no sounds were played. Furthermore, there was no correlation between the metacognitive judgments and the objective sound effects within any of the conditions (all *r’*s < .07).^1^

### Discussion

In line with the pattern of findings observed in previous studies (Bell, Mieth, et al., 2019; Hughes et al., 2007; Marsh et al., 2020), the objective measures of distraction showed evidence of both an auditory-deviant effect and a changing-state effect, with changing-state sequences being more disruptive to serial recall than auditory-deviant sequences. Of interest was whether the prospective and retrospective metacognitive judgments about the effects of the auditory word sequences would correspond to, or dissociate from, these objectively measured effects. The findings displayed in Figs. 3 and 5 show that, on average, the subjective metacognitive judgments mapped well onto the objective performance pattern displayed in Fig. 4. The disruptive effect of changing-state relative to steady-state sequences was thus, on average, well reflected in the metacognitive judgments. What is more, participants were able to verbalize the experienced disruption by changing-state sequences in an open-answer format. The results thus disconfirm the postulated hypothesis that the changing-state effect is, in contrast to the auditory-deviant effect, based on introspectively inaccessible processes.

It is only the degree to which an individual participant is distracted more or less than other participants that is not well reflected in individual differences in the metacognitive judgments. Even though, on average, the relative disruptive effect of steady-state, auditory-deviant, and changing-state sequences were correctly assessed, those participants who thought that they were – relative to other participants – less negatively affected by the sounds were not necessarily those who were actually less negatively affected. This applied equally to the disruptive effects of auditory-deviant and changing-state sequences.

## General discussion

The present study served to test the prediction derived from the duplex-mechanism account of auditory distraction (Hughes & Marsh, 2019) that people are aware of the disruptive effect of deviant stimuli but are unaware of the detrimental effect of changing-state sequences on serial recall because only the former, not the latter, is caused by a diversion of focused attention. The present results disconfirm this prediction.

When judging the effect of sound sequences that were only verbally described, participants expressed the abstract belief that both auditory-deviant sequences and changing-state sequences had a more pronounced disruptive effect on serial recall than steady-state sequences. Auditory-deviant sequences were believed to be as disruptive as changing-state sequences. At first glance, the disruptive effects of auditory deviants seem to have been overestimated relative to their comparatively small objective effects on performance. However, the pattern of results cannot count as a confirmation of the hypothesis that the changing-state effect is based on automatic processes that occur outside of, and are inaccessible to, people’s awareness, which implies that people “show little or no subjective awareness” of this effect (Hughes & Marsh, 2019, p. 138). When metacognitive judgments were based on the perception of specific sound sequences, participants were, on average, well able to judge the relative disruptive potential of steady-state, auditory-deviant, and changing-state sequences. Specifically, the pattern of the prospective metacognitive judgments (Fig. 3) corresponds very well to the actual effects of steady-state, auditory-deviant, and changing-state sequences on performance (Fig. 4). In addition, the strong disruptive effect of the changing-state sequences experienced during the serial-recall task were quite accurately reflected in the retrospective metacognitive judgments (Fig. 5) and were clearly expressed in plain language (Fig. 6). The present results thus show that people are well aware of the disruptive potential of changing-state sequences. This complicates the classification of the changing-state effect as being generated by metacognitively inaccessible “Type I” processes according to the classification scheme displayed in Table 1.

The present results are evidence against the dissimilarity between the two classes of distraction postulated by the duplex-mechanism account (Hughes, 2014). Given the novelty of the hypothesis that the changing-state effect and the auditory-deviant effect are dissociated by awareness (Hughes & Marsh, 2019), one may argue that the refutation of the postulated dissociation leaves the key components of that account intact. However, such accounts are not only evaluated by how well they can explain established findings but also by how well they predict new findings. The results presented here reduce the appeal of the idea of a strict dichotomy between attention-based, metacognitively accessible interference on the one side and metacognitively inaccessible interference-by-process on the other. Furthermore, the present findings add to a growing body of evidence that casts doubt on whether phenomena of auditory distraction reflect two fundamental classes that can be dissociated by a set of dichotomous characteristics. Specifically, the results of the present study align well with those of previous studies that provide evidence against dissociations between the auditory-deviant effect and the changing-state effect in cognitive control, domain specificity, and dependence on general cognitive resources (Bell et al., 2013, 2017, in press; Körner et al., 2017, 2019; Röer et al., 2018; Röer, Bell, Marsh, & Buchner, 2015).

The aim of the present study was to provide an empirical test of the hypothesis that people should be aware of the auditory-deviant effect but unaware of the changing-state effect. Based on the results, this hypothesis can be rejected: There is no conclusive evidence for a clear dissociation in metacognitive awareness between the auditory-deviant effect and the changing-state effect. However, the present study gives the opportunity to raise more general questions about the metacognition of auditory distraction, such as: How do people arrive at metacognitive judgments about the effect of background sounds on cognitive performance? Do they have partial or full access to the processes underlying distraction or are their judgments based on simple heuristics? Are they able to reliably predict the disruptive effect of task-irrelevant sound on cognitive performance in everyday life? These general questions are more difficult to answer because they require a more comprehensive approach than the focused research question addressed here. The present study can be useful in generating new hypotheses and by providing a new methodological approach that can be used to examine these hypotheses, but further studies are needed to explicitly address the generality of the findings. As a start, an interesting observation is that metacognitive beliefs that were made in the absence of prior exposition to the sounds (Fig. 2) reflected the actual effects of the sound sequences on performance (Fig. 4) less accurately than the prospective and retrospective judgments (Figs. 3 and 5). This may indicate that the prior exposition to the sounds may have an important influence on the validity of the metacognitive judgments. Given that both the prospective and the retrospective judgments accurately reflected the effects of the sound sequences that were objectively measured, one may be tempted to conclude that people have a direct access to the processes that are responsible for the performance decrement. While it does, indeed, seem possible that people have direct access to the degree to which auditory-deviant and changing-state sounds capture their attention, it also seems possible that participants rely on heuristic cues such as a feeling of ease or difficulty (Alter & Oppenheimer, 2009). For example, participants may judge that a constantly repeated and thus easy-to-process stimulus is unlikely to draw resources away from a primary task. The degree to which the cues underlying the metacognitive judgments are reliably correlated with distraction and, thus, lead to correct judgments about distraction across different contexts is, ultimately, an empirical question (cf. Begg et al., 1989; Koriat, 1997). The conclusion that people are aware of the disruptive effect of changing-state sequences simply refers to the fact that participants were able to ascertain that changing-state sequences had a detrimental effect on serial recall. However, it would be premature to conclude that participants have direct insight into the mechanisms underlying auditory distraction. To arrive at such a broad conclusion, it would be necessary to systematically evaluate the accuracy of metacognitive judgments of distraction across different types of stimuli and contexts, which is beyond the scope of the present paper.

On the face of it, the finding that there is no correlation between the prospective and retrospective metacognitive judgments and objectively measured distraction at an individual level (Fig. 7) may indicate that metacognitive insight into auditory distraction is limited. Whether people judged the sounds to have beneficial or detrimental effects on their performance was completely unrelated to the size of the distraction effect that was objectively measured. From this finding in isolation, one may be inclined to conclude that people cannot accurately judge the effects of different types of sounds on their performance. However, these findings simply show that it is much more difficult to judge the absolute level of one’s distraction than to judge the relative differences in the effects of different types of stimuli on one’s performance that are directly contrasted with each other. Furthermore, before generalizing this finding, it is worth noting that standard effects of auditory distraction that aim at isolating theoretically interesting factors are of a smaller order of magnitude than naturalistic sounds such as music and continuous background speech (e.g., Bell et al., 2017; Röer et al., 2014). It thus remains to be tested in future studies whether people are better at evaluating the effects of naturalistic background sounds that are often experienced and have a stronger effect on performance.

In summary, the present study served to test a novel prediction derived from the duplex-mechanism account, namely that people are aware of the disruption of performance by deviant sounds but have little to no awareness of the disruptive effect of changing-state sounds on serial recall (Hughes & Marsh, 2019). This hypothesis was disconfirmed by the present results. Participants were, on average, well aware of the greater disruptive effects of auditory-deviant and changing-state sequences relative to those of steady-state sequences. The present findings thus indicate that phenomena of auditory distraction cannot be categorized in two separate classes based on their relative accessibility to awareness.

## Footnotes

1 The same is true when metacognitive judgments about the auditory-deviant effect (i.e., the difference between the steady-state condition and the auditory-deviant condition) and the changing-state effect (i.e., the difference between the steady-state condition and the changing-state condition) are directly contrasted with the auditory-deviant effect and the changing-state effect (measured by the difference between the steady-state condition and the auditory-deviant condition and by the difference between the steady-state condition and the changing-state condition), respectively (all *r*s ≤ .13).
